# Supplementary material for: Multi‐dataset identification of innovative feature genes and molecular mechanisms in keratoconus
Source: J Cell Mol Med. 2024 Sep 19;28(18):e70079. doi: 10.1111/jcmm.70079 (PMC11412914; doi:10.1111/jcmm.70079)
Supplement: Supplementary file 2 — Material S2. [file JCMM-28-e70079-s002.zip › 7.GSEA analysis/1.ARL11/KEGG_THYROID_CANCER.html]

Details for gene set KEGG\_THYROID\_CANCER[GSEA]

|  || Dataset | ARL11.group.cls#h\_versus\_l |
| Phenotype | group.cls#h\_versus\_l |
| Upregulated in class | l |
| GeneSet | KEGG\_THYROID\_CANCER |
| Enrichment Score (ES) | -0.28593132 |
| Normalized Enrichment Score (NES) | -0.9923845 |
| Nominal p-value | 0.47843942 |
| FDR q-value | 1.0 |
| FWER p-Value | 1.0 |
Table: GSEA Results Summary

  

Fig 1: Enrichment plot: KEGG\_THYROID\_CANCER      
 Profile of the Running ES Score & Positions of GeneSet Members on the Rank Ordered List

  

| SYMBOL | TITLE | RANK IN GENE LIST | RANK METRIC SCORE | RUNNING ES | CORE ENRICHMENT || 1 | RET | na | 2305 | 0.276 | 0.0482 | No |
| 2 | NTRK1 | na | 4617 | 0.179 | 0.0557 | No |
| 3 | TP53 | na | 9447 | 0.068 | -0.0553 | No |
| 4 | RXRA | na | 9938 | 0.061 | -0.0441 | No |
| 5 | MYC | na | 10444 | 0.054 | -0.0361 | No |
| 6 | BRAF | na | 10628 | 0.052 | -0.0197 | No |
| 7 | KRAS | na | 10630 | 0.052 | 0.0019 | No |
| 8 | TCF7L2 | na | 10815 | 0.050 | 0.0172 | No |
| 9 | CTNNB1 | na | 14653 | 0.014 | -0.0876 | No |
| 10 | CCDC6 | na | 16222 | 0.002 | -0.1319 | No |
| 11 | CCND1 | na | 16546 | -0.000 | -0.1412 | No |
| 12 | CDH1 | na | 16747 | -0.002 | -0.1463 | No |
| 13 | MAP2K2 | na | 16997 | -0.003 | -0.1521 | No |
| 14 | NCOA4 | na | 17320 | -0.006 | -0.1591 | No |
| 15 | TCF7 | na | 18185 | -0.012 | -0.1792 | No |
| 16 | TPM3 | na | 18375 | -0.013 | -0.1793 | No |
| 17 | HRAS | na | 18435 | -0.013 | -0.1755 | No |
| 18 | RXRB | na | 22268 | -0.043 | -0.2683 | Yes |
| 19 | MAPK3 | na | 22614 | -0.046 | -0.2590 | Yes |
| 20 | PAX8 | na | 22674 | -0.047 | -0.2412 | Yes |
| 21 | TFG | na | 23494 | -0.056 | -0.2417 | Yes |
| 22 | TCF7L1 | na | 24530 | -0.070 | -0.2426 | Yes |
| 23 | MAPK1 | na | 24626 | -0.071 | -0.2158 | Yes |
| 24 | TPR | na | 26694 | -0.102 | -0.2329 | Yes |
| 25 | NRAS | na | 27790 | -0.126 | -0.2124 | Yes |
| 26 | PPARG | na | 28947 | -0.155 | -0.1813 | Yes |
| 27 | MAP2K1 | na | 31070 | -0.223 | -0.1500 | Yes |
| 28 | LEF1 | na | 32575 | -0.292 | -0.0720 | Yes |
| 29 | RXRG | na | 33017 | -0.323 | 0.0493 | Yes |
Table: GSEA details [plain text format]

  

Fig 2: KEGG\_THYROID\_CANCER      
 Blue-Pink O' Gram in the Space of the Analyzed GeneSet

  

Fig 3: KEGG\_THYROID\_CANCER: Random ES distribution      
 Gene set null distribution of ES for **KEGG\_THYROID\_CANCER**
